# Determining the transformative potential of circular agriculture initiatives

**DOI:** 10.1007/s13280-023-01894-5

**Published:** 2023-07-13

**Authors:** Jelle Silvius, Anne G. Hoogstra, Jeroen J. L. Candel, Evelien M. de Olde, Imke J. M. de Boer, Catrien J. A. M. Termeer

**Affiliations:** 1 Public Administration and Policy Group, Department of Social Sciences, Wageningen University & Research, 6700 EW Wageningen, The Netherlands; 2 Animal Production Systems, Department of Animal Sciences, Wageningen University & Research, 6700 AH Wageningen, The Netherlands

**Keywords:** Circular agriculture, Transformation, Transformative potential, Transition, Transition initiatives

## Abstract

Policymakers and scientists regard emerging circular initiatives as levers for transformations towards more sustainable food systems. However, it remains unclear how to determine the extent to which circular initiatives have transformative potential. That is, can these initiatives foster a transformation as a result of how they currently bring circularity into practice? In the transformation literature, the characteristics of transformative initiatives are conceptualised in a generic and abstract way. To address this gap, we develop a heuristic of five characteristics for potentially transformative circular agriculture initiatives, which we illustrate with examples of existing initiatives. The heuristic builds on the ‘small wins’ and circular agriculture literature. Initiatives that hold transformative potential contribute to circular agriculture principles with outcomes that are concrete, in-depth and both technological and social in nature. Additionally, these initiatives faced barriers and overcame them. The heuristic enables policymakers, who call for circular solutions, to identify truly transformative circular initiatives.

## Introduction

Current global food production and consumption are responsible for a substantial share of greenhouse gas emissions, eutrophication, land use change and biodiversity decline, threatening not only the prospects for food production itself but the general liveability of the planet (Conijn et al. 2018; Rockström et al. 2020). Moreover, global food production largely relies on inputs that are finite and increasingly scarce, such as phosphate and fossil fuels (Stamm et al. 2022).A shift towards alternative modes of food production and consumption that are less polluting and resource consuming is needed. Circular agriculture is proposed as such an alternative (Jurgilevich et al. 2016; Van Zanten et al. 2019; Koppelmäki et al. 2021). Based on the principles for a circular bioeconomy (Muscat et al. 2021), we define circular agriculture as a way of producing and consuming food that safeguards the health of agro-ecosystems, prevents losses and waste of biomass and nutrients, reuses and recycles unavoidable residual streams in an efficient way and minimizes the use of energy.

Circularity is also at the core of the European Union (EU) Farm-to-Fork strategy, which aims to *‘reduce nutrient losses by 50%’* and to use the opportunities of the *‘circular bio-based economy’* in farming (European Commission 2020). The strategy regards innovation, experiments and behavioural changes as *‘key drivers’* for change and calls for *‘innovative solutions’* aimed at circularity (European Commission 2020). The literature on circular agriculture also acknowledges the importance of stimulating emerging circular initiatives, as these initiatives may be the starting point for a larger transformation (Jurgilevich et al. 2016; Muscat et al. 2021; Valencia et al. 2022). We conceptualise these circular initiatives as emerging, new ways of ‘thinking, doing and organising’ (Gorissen et al. 2018) aimed at increasing circularity in agriculture. Examples include new farm models, such as ‘Kipster’, a circular laying hen farm in which the hens are fed leftovers from the food industry, or community-supported agriculture, such as the initiative of ‘Herenboeren’, a cooperation of producers and consumers who strive for sustainable food production (Schagen et al. 2022).

However, it remains unclear to what extent emerging circular initiatives are actually transforming rather than optimising the current agricultural system. A shift towards circular agriculture cannot be achieved by mere improvements within the current system. Instead, realising circular agriculture implies an ongoing process of fundamental changes in practices, organisations, markets and institutions (Muscat et al. 2021). This process of profound, systemic change is also referred to as a ‘transition’ or ‘transformation’ (Feola 2015; Köhler et al. 2019). However, as the concept of circular agriculture is still rather ambiguous, there is a risk that initiatives aimed at circularity remain superficial, thereby undermining the concept’s promise for fundamental change (Kirchherr et al. 2017). Since policymakers aim to support these initiatives, it is important to be able to recognise the extent to which they are truly transformative.

This practical challenge resonates with a theoretical gap in the transformation literature. Various transformation studies refer to existing initiatives that may have the potential to scale up and ultimately foster transformations towards more desirable futures (Gorissen et al. 2018; Lam et al. 2022). Such initiatives are referred to with terms like ‘niches’ (Smith and Raven 2012), ‘transition initiatives’ (Gorissen et al. 2018), ‘transformative innovation’ (Loorbach et al. 2020), ‘small wins’ (Termeer and Metze 2019) or ‘seeds of a good Anthropocene’ (Bennett et al. 2016). However, as we argue in "Current conceptualisations of transformative initiatives" section of this paper, the conceptualised characteristics of these terms are not sufficiently well-defined to actually recognise and distinguish potentially transformative initiatives from less significant ones. Studies typically focus on the scaling of initiatives, but do not question what types of initiatives are to be supported to achieve the desired transformative impact (El Bilali 2019). Moreover, current conceptualisations of initiatives do not relate their characteristics to particular desired directions of change. In order to recognise transformative circular initiatives, initiatives’ characteristics need to be specified according to the ambition of circularity.

Whereas we acknowledge that it remains impossible to know beforehand whether an initiative will ultimately be transformative (Hebinck et al. 2021), initiatives may carry a certain promise to do better, their eventual impact depending on the extent to which initiatives develop and grow over time (Van der Ploeg and Wiskerke 2004). Scholars have theorised various ‘amplification mechanisms’ through which the impact of initiatives may grow, which remains an inherently uncertain and complex process (Lam et al. 2020). This paper starts from the presumption that despite these challenges, initiatives may have certain characteristics that indicate their ‘transformative potential’ (Hebinck et al. 2021). We understand transformative potential as an initiatives’ plausible contribution to a transformation towards circular agriculture, as a result of an amplification of how the initiative currently brings circularity into practice. To assess the latter, we conceptualise a number of characteristics that define initiatives’ current contribution to circular agriculture.

The question we address in this paper is: *what characteristics determine the transformative potential of circular agriculture initiatives?* To address this question, we develop a heuristic of five characteristics to reflect upon the transformative potential of circular agriculture initiatives. The heuristic builds on the concept of small wins, which unlike other conceptualisations of initiatives provides five general characteristics for transformative initiatives (Termeer and Metze 2019). To specify these general characteristics to the ambition of circular agriculture, we connect these characteristics to five principles for circularity as defined by Muscat et al. (2021) which we translate to the context of agriculture. We apply the heuristic to three examples of circular initiatives to demonstrate its application.

The paper is structured as follows: section two provides an overview and comparison of current conceptualizations of transformative initiatives. We conclude that the concept of small wins offers the most explicit conceptualization of characteristics. In section three, we further explain the five characteristics of small wins and the circular agriculture principles. Connecting these building blocks, we present the heuristic in section four, while section five discusses its scientific and practical implications.

## Current conceptualisations of transformative initiatives

In response to the urgency of today’s global environmental challenges, the topic of ‘transitions’ or ‘transformations’ and how such processes of change can be stimulated is increasingly studied (Feola 2015; Köhler et al. 2019). While the field of transformation and transition studies initially engaged with historic cases of societal transformations, such as the modernisation of agriculture (Grin 2012), it has increasingly become more focused on the drivers for future sustainability transformations (Köhler et al. 2019).Various transformation theories pose that present, small-scale initiatives may be levers for larger, systemic change. These initiatives are referred to with different terms, see Table 1.

**Table 1:** Conceptualisations of initiatives as seeds for transformations

Term	Conceptualisation	Sources
Niche-innovations	*‘Technological niches form the micro-level where radical novelties emerge. These novelties are initially unstable sociotechnical configurations with low performance. Hence, niches act as ‘incubation rooms’ protecting novelties against mainstream market selection. Niche-innovations are carried and developed by small networks of dedicated actors, often outsiders or fringe actors.’*	Geels (2002), Schot and Geels (2008), and Smith and Raven (2012)
Transition initiatives	*‘Actor networks that start-up, adopt and/or engage with new practices, technologies and experiments that seek to profoundly change established unsustainable routines and perceptions towards more sustainable ones.’*	Gorissen et al. (2018)
Transformative innovations	*‘Shared activities, ideas and objects across locally rooted sustainability initiatives that explore and develop alternatives to incumbent and (perceived) unsustainable regimes that they seek to challenge, alter or replace.’*	Loorbach et al. (2020)
Seeds of a good anthropocene	*‘Seeds are initiatives (social, technological, economic, or social–ecological ways of thinking or doing) that exist, at least in prototype form, and that represent a diversity of worldviews, values, and regions, but are not currently dominant or prominent in the world.’ Seeds ‘improve social, ecological, or economic dynamics within a particular setting’*	Bennett et al. (2016)
Grassroots innovations	*‘Networks of activists and organisations generating novel bottom–up solutions for sustainable development; solutions that respond to the local situation and the interests and values of the communities involved. In contrast to mainstream business greening, grassroots initiatives operate in civil society arenas and involve committed activists experimenting with social innovations as well as using greener technologies.’*	Seyfang and Smith (2007)
Small wins	*‘Concrete, in-depth changes, which get reinforced over time and accumulate into transformative change through non-linear mechanisms’*	Termeer and Dewulf (2019), Termeer and Metze (2019), and Bours et al. (2021)

Although many scholars acknowledge the potential of initiatives to foster transformations, their conceptualisations give little guidance on how to actually recognise these initiatives in practice. For example, the term ‘niche’ is embedded in the multi-level perspective that theorises transformations as an interaction between initially unstable initiatives that compete with dominant practices in the ‘regime’ (Geels 2002). The concept emphasises the initiative’s initial low performance as compared to dominant practices, its need for protection from regime influences and its initiation by dedicated actors from outside the regime (Smith and Raven 2012). The distinction to the regime is also central in the conceptualisation of ‘transformative innovation’, which ‘aims to challenge or to replace dominant regime practices’ (Loorbach et al. 2020). Other terms also relate initiatives to specific types of actors, such as in ‘civil society arenas’, ‘committed activists’ (Seyfang and Smith 2007) or ‘actor networks’ (Gorissen et al. 2018). For these conceptualisations, the focus is on the place of emergence (i.e. niche or regime) or its relation to particular actors (e.g. activists), while largely ignoring the specific outcomes that would make the initiative transformative. Moreover, the analytical distinction between niche and regime is not as clear-cut in practice, notably in agricultural transformations, which makes it hard to distinguish niche initiatives empirically (El Bilali 2019). Other terms such as ‘seeds of a good Anthropocene’ (Bennett et al. 2016) and ‘transition initiatives’ (Gorissen et al. 2018) put more emphasis on the outcomes of the initiative, by characterising them as ‘improving conditions’ and profoundly changing unsustainable practices. There seems to be a shared recognition among all terms that promising initiatives have a radical nature. However, the general characteristics of initiatives remain relatively abstract.

To overcome these shortcomings of current conceptualisations of initiatives, we build the heuristic on the concept of small wins, which unlike other conceptualisations of transformative initiatives, provides five explicit characteristics to identify small wins (Termeer and Metze 2019). Additionally, by providing five general characteristics for transformative initiatives, small wins acknowledges that significant steps in transformation processes may emerge in all parts of society, including organizations that are associated with the regime.

## Conceptual building blocks

Here, we present the two existing frameworks that we build on and connect in "Heuristic for determining the transformative potential of circular agriculture initiatives" section to develop the heuristic.

### Small wins

Originally defined in organizational science (Weick 1984) and more recently applied to the governance of transformations (Termeer and Dewulf 2019; Bours et al. 2021), small wins are concrete, in-depth changes that contribute to a societal objective and may accumulate into transformative change through reinforcing mechanisms. Small wins theory is rooted in theories of incrementalism (Lindblom 1979) and continuous change (Weick and Quinn 1999), assuming that actors throughout society continuously react, adapt and improvise to societal challenges with small, but meaningful and in-depth steps, or small wins. A transformation can be steered to a certain extent by identifying, appreciating and supporting small wins. Termeer and Metze (2019) develop a framework for the governance of transformations, which prescribes three steps: (1) setting a provocative ambition (2) identifying small wins that contribute to this ambition (3) stimulating the mechanisms through which small wins amplify their impact (Termeer and Metze 2019). To identify small wins, Termeer and Metze (2019) conceptualize five important characteristics of small wins. First, small wins have concrete, completed outcomes, going beyond promises or mere ideas. Second, the initiative makes a clear and demonstrated contribution to a particular societal objective, such as circularity. Third, small wins are not quick fixes or low-hanging fruit but entail in-depth changes. This means that small wins break with existing routines, beliefs, mindsets and operational logics that define prevailing ways of doing things. Fourth, because small wins face tensions with the current system, the initiatives inevitably face barriers that they need to overcome by creating more favourable conditions, seizing opportunities or altering constraints. Fifth, small wins have elements of both technological and social change, which strengthen each other.

These characteristics offer a basis to define what constitutes the transformative potential of circular agriculture initiatives. However, some of the characteristics are still defined at a relatively high level of abstraction and remain generic. The characteristics require further specification to the relevant context, e.g. to know what constitutes in-depth change and a meaningful contribution to a transformation to circular agriculture. Besides, the characteristics by Termeer and Metze (2019) are presented as binary criteria. In practice, however, initiatives may comply with the characteristics to greater and lesser extents. To reflect on an initiative’s transformative potential, the question is not whether it qualifies as a small win but to what extent it meets the small win characteristics. We therefore conceptualize different levels of transformative potential for each characteristic.

### Circularity principles

The concept of circular agriculture derives from that of the circular economy, referring to an *‘economic system that replaces the ‘end-of-life’ concept with reducing, alternatively reusing, recycling and recovering materials in production/distribution and consumption processes’* (Kirchherr et al. 2017). The current global food system is characterised by a great dependence on external inputs such as synthetic fertilisers, an increasing decoupling of crop- and livestock farming systems leading to local nutrient surpluses or shortages (Schulte-Uebbing et al. 2022), and a waste of materials and nutrients through leakages and losses such as eutrophication to the environment, food waste and the loss of human excreta (Jurgilevich et al. 2016). The premise of circular agriculture is to replace this extractive and wasteful model with a model in which nutrients and materials are continuously circulated, losses and waste are prevented and natural resources are used more efficiently to feed a growing population (Jurgilevich et al. 2016; Van Zanten et al. 2019; Koppelmäki et al. 2021). The latter also implies that land and biomass should primarily be used to feed humans, instead of farm animals (Van Zanten et al. 2019).Circular agriculture shows overlap with other directions of change such as agro-ecology and regenerative agriculture as it shares the aim of reducing the environmental impact of food production, but puts more emphasis on the efficient use and circulation of biomass and nutrients to reach this aim (see Vermunt et al. 2022).

There are some challenges when it comes to assessing whether an initiative contributes to circular agriculture. Like the concept of circular economy (Kirchherr et al. 2017), the definition of circular agriculture remains ambiguous (Dagevos and de Lauwere 2021). Besides, contributing to circular agriculture is not a one-dimensional objective - like reducing emissions — but implies integrating several principles related to the management of, e.g. the soil, natural resources, energy and biodiversity in agricultural practices. This implies that a contribution to circular agriculture cannot be measured using single indicators. Moreover, an important feature of transformations is that the end-state is partly unknown and ambiguous to attract the support of a wide range of actors and to keep the solution space open (Candel 2022). To overcome this tension between the inevitable ambiguity of transformation objectives on the one hand and the need to define which actions contribute to these objectives on the other, we build our heuristic on five principles, developed by Muscat et al (2021). These principles are aimed to guide biomass use towards circularity, while they leave enough room for various interpretations on how this can be done. In Table 2, we present the five principles and explain their implications for agriculture.

**Table 2:** Circular agriculture principles

Principles	Explanation	Implications for agriculture
1. **Safeguard** the health of our agro-ecosystems	Ecosystem stocks (like soils and forests) must not be used beyond their regenerative capacity and sinks (like the air) must not be polluted	Avoid emissions to the environment (e.g. CO_2_, N, P) Cease the use of finite resources such as fossil fuels, phosphate rock Protect and regenerate biodiversity and soil health
2. **Avoid** the production of non-essential products and the waste of essential resources	Because materials can never be fully recycled and production processes inevitably create pollution, unnecessary production and losses must be prevented	Prevent food waste and overconsumption Prevent nutrient losses Prevent resource-intensive inputs like synthetic fertiliser
3. **Prioritise** the use of natural resources for basic human needs	Natural resources like biomass and land are scarce and should be used effectively. This implies that biomass should be used for essential needs first	Land and biomass should be used primarily to feed humans instead of farm animals Farm animals should only be fed non-human edible biomass
4. **Recycle** by-products of the agro-ecosystem	Even if losses and waste are avoided (principle 2), the production of food comes with residual streams such as manure and crop residues. These should be reused in the food system at their highest utility	Use residual streams to feed farm animals, to fertilise soils and to produce biomaterials Only use materials that are not safe for recycling to produce energy
5. **Entropy,** or minimise and avoid the use of energy and use renewable energy sources	Recycling of nutrients and materials inevitably costs energy, therefore energy use should be minimised in addition to transitioning to renewables	Minimise and avoid energy use Use renewable energy sources

Based on Muscat et al. (2021)

## Heuristic for determining the transformative potential of circular agriculture initiatives

**Fig. 1:**
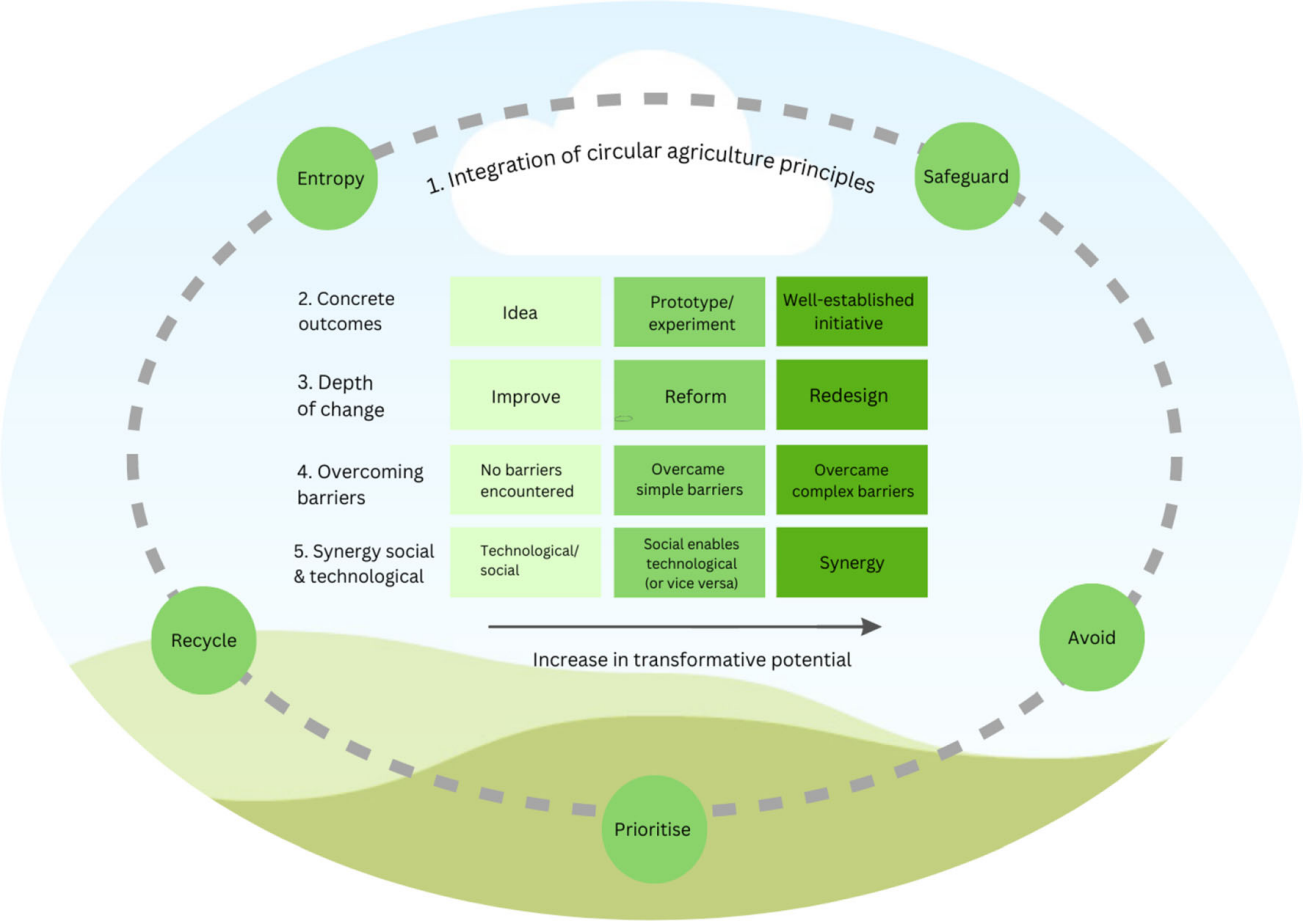
The five characteristics that determine the transformative potential of circular agriculture initiatives. Initiatives may exhibit these characteristics to different extents, indicating different levels of transformative potential

Connecting the principles for circular agriculture to the five characteristics for small wins, we conceptualise a heuristic of five characteristics which define the transformative potential of circular agriculture initiatives (Fig. 1). We propose that initiatives may contribute to circular agriculture at different levels of transformative potential, depending on the extent to which initiatives meet the small wins characteristics. Below, we further conceptualise each characteristic and its levels, and demonstrate the varying transformative potential of existing examples of circular agriculture initiatives (as presented in Box 1).

### Integration of circular agriculture principles

In order to have transformative potential in a transformation to circular agriculture, an initiative should have a clear and demonstrated contribution to circular agriculture. An initiative’s contribution to circular agriculture is determined by the extent to which it addresses and integrates the five circular agriculture principles (as presented in Table 2). As all principles are of equal importance for circular agriculture, there is no hierarchy between the principles. An initiative does not necessarily have to address all principles and may have transformative potential if it addresses only one. However, as circular agriculture is supposed to be an integral solution to environmental challenges, trade-offs between principles should be avoided as much as possible. For example, waste streams should be prevented (principle 2) before these are recycled (principle 4).

The illustrative initiatives (as described in Box 1) address the principles in different ways. *Fertile Nutrient Cycles* contributes to the safeguard principle through the reduction of N and P emissions by measures such as an improved timing of the fertilisation that better meets the needs of the crops and by mixing manure with water in the fertilisation process. Because these measures avoid the losses of valuable nutrients and thereby increase resource efficiency the initiative also contributes to the avoid principle. The prioritise principle is addressed to a limited extent because farmers in the project are encouraged to reduce the use of feed concentrates that compete with resources for human edible biomass. *Circular Broilers* mainly addresses the recycle principle by feeding broilers residual streams from arable farming and returning manure to the arable land. The initiative addresses the prioritise principle as the feed for the broilers is replaced by human inedible residual streams. Through production of renewable energy, the initiative contributes to the entropy principle. By composting municipal waste streams and using the compost as a fertiliser to substitute artificial fertiliser, *Agricycling* contributes not only to the recycle principle but also to the avoid and safeguard principle. The use of artificial fertiliser, of which the production consumes fossil fuels, is avoided because it is replaced using the nutrients from municipal waste streams. Because these waste streams contain organic carbon, the use of the compost sequesters soil organic carbon, having a positive impact on the carbon cycle and soil life.

### Concrete outcomes

To have transformative potential, an initiative should go beyond mere promises or ideas to implement circularity. The initiative should generate concrete outcomes that improve conditions for actors involved. We follow Benett et al. (2016) who define ‘seeds of a good anthropocene’ as being at least a prototype, experiment or start-up. All three of the initiatives are well-established initiatives, showing concrete outcomes for participants and other stakeholders.

### The depth of change

Initiatives may address the circular agriculture principles at different levels of change, for example by adjusting conventional practices or by introducing completely new ones. More profound changes that address the causes of problems at a deeper level have more transformative potential (Abson et al. 2017). To conceptualise the depth of change, we distinguish between three orders of change: first order (optimise), second order (reform) and third order (redesign) (Table 3). These three orders resonate with different conceptualisations of change as in the policy sciences (Hall 1993) and organisational sciences (Argyris and Schon 1978). We build on these conceptualisations and the work on processes of change by Pahl-Wostl (2009) and Gliessmann (2016) to define different levels of change in the context of circular agriculture:

1. **First order change (optimise)** refers to improvements or refinements of practices and techniques within the existing mind-set. That is, the goals and the guiding assumptions on how these goals can be achieved remain unchanged (Pahl-Wostl 2009). These improvements typically aim to reduce resource use and the negative impact to the environment by making the use of conventional inputs and practices more efficient or less harmful (Gliessman 2016). It may also include additional measures (like flower strips in field margins) that are possible without a significant change of established practices.
2. **Second order change (reform)** breaks through the prevailing mind-set by a reflection and reframing of how problems are approached and how goals can be achieved. This results in novel practices and techniques and may involve new relationships that open up the possibilities for solving problems (Pahl-Wostl 2009). In the context of circular agriculture, it may imply that resources are circulated between parties or that alternative practices that are less resource-consuming are adopted. However, the main structure and functions of the agricultural system remain largely unchanged (Gliessman 2016).
3. **Third order change (redesign)** refers to a significant change of the values, goals and identities that underpin the agricultural system. These changes result from a recognition that problems are inherent to the linear system design and its prevailing goals, or the intent of the system (Abson et al. 2017). The aim is to reconsider the design of the system to address the root causes of problems, or ‘to prevent problems before they occur, rather than trying to control them after they happened’ (Gliessmann 2016). It may imply new ways to organise the flows of natural resources, more diverse functions and revisited boundaries of the agricultural system.

**Table 3:** Overview of the illustrative initiatives and their transformative potential

Initiative	Characteristics				
	Integration of circularity principles	Concrete outcomes	Depth of change	Overcoming barriers	Synergy social and technological change
Fertile nutrient cycles	Safeguard (emission reduction), Avoid (better manure efficiency), Prioritise (less feed concentrates)	Yes	First order change: Mainly improvements within the logics of the current linear system	No serious barriers encountered. Efficiency improvements benefited farmers economically	Primarily technological changes. Knowledge sharing enables diffusion
Circular broilers	Recycle (residual streams as feed), Prioritise (no human edible feed concentrates), Entropy (renewable energy production)	Yes	Second order change: Breaks through the existing mindset of linear specialisation by integrating functions of arable and livestock farming. Functions themselves remain unchanged	Overcame economic barriers by cooperating with a meal box company to compensate higher operation costs	Primarily technological, enabled by novel cooperations with neighbouring farmers and a meal box company
Agricycling	Recycle (municipal waste streams as fertiliser), Avoid (no synthetic fertiliser), Safeguard (sequestering carbon and improving soil health)	Yes	Third order change: Redesign of the structure of nutrient flows by reusing municipal waste streams, reconnecting agriculture with society. Farmers become waste-up-cyclers besides their traditional role as food producers	Overcame hard institutional barriers. Exemptions granted by local officials for waste management regulations	Both the technological and social change are central and strengthen each other. Recycling is a new revenue model and regulatory exemptions enable replication in other areas

Each of the example initiatives represents a different level of change. In the case of *Fertile Nutrient Cycles*, the encouraged techniques are first order changes based on refining and adjusting existing practices and techniques. For example, farmers are encouraged to refine the timing of manure application and to add water to the manure while spreading it on the land to increase the manure efficiency and avoid emissions. Although these techniques are improvements, the practices themselves remain largely unchanged. The project is best characterised as an optimisation of the current system, as the linear logic of using resource-intensive inputs to achieve high outputs is hardly questioned.

The initiative of *Circular Broilers* is an example of a second order change as it represents a shift in the prevailing assumptions on how to produce food. Whereas animal and arable farming have been specialised and separated over the past decades (Garrett et al. 2020), encouraged through the availability of imported feed concentrates and synthetic fertilisers, this initiative integrates both sectors. The aim is to reduce the resource use of the two sectors through cooperation and the recycling of residual streams. The initiative shows an integration of functions, which themselves however remain largely unchanged. *Circular Broilers* therefore represents a reform of the agricultural system.

*Agricycling* could be characterised as a third order change. This initiative is rooted in the idea that many of modern agriculture’s problems occur due to a separation between agriculture and society. The initiative therefore reconnects agriculture with society through the reuse of municipal residual streams and aims to ultimately deepen this by reusing human excreta. The structure of the flows of natural resources in the agricultural system and its connection to other systems are reconsidered and redesigned. The farmers within the cooperative take on a new identity as waste up-cyclers besides their traditional role as food producers. This new identity is confirmed by the institutional barriers this initiative had to overcome, as the cooperative does not fit current legal frameworks for waste management.

The examples illustrate that although initiatives may contribute to the same circular principles, they may do so at different levels of change. While improvements within the current system (first order changes) may be beneficial for ecological conditions, true transformations towards circularity require the recognition that problems of transgressing ecological boundaries are inherent to current system design. The deeper the level of change, the more problems are addressed at their root cause. First-order changes on the other hand may be easier to achieve and may be a first step for actors to put circular principles into practice.

### Overcoming barriers

Because transformative initiatives challenge the logics and assumptions of the current linear system, they inevitably face institutional, economic, cultural or technological barriers. Termeer and Metze (2019) even state that *‘if a small step is realised smoothly and without resistance, it may not be an in-depth change’*. The fourth characteristic therefore relates to the initiative having overcome one or more barriers by circumventing these barriers or by creating conditions to cope with these. Overcoming barriers makes initiatives more transformative as other actors may learn from the ways in which these initiatives have dealt with the barriers, a mechanism known as ‘learning-by-doing’ (Termeer and Metze 2019). We follow Biesbroek et al. (2013) in defining barriers as *‘the actors’ subjective interpretations or collective understanding of factors and conditions that emerge from the actors, the governance system or the system of concern, which the actor values as having a negative influence on the process and reduce the chances of successful outputs, but that are manageable and can be overcome with concerted efforts, or by creating and seizing opportunities’*. The extent to which barriers are perceived is subjective, but in the transition to circular agriculture we can identify certain barriers that are collectively experienced (Biesbroek et al. 2013). These barriers may be technological (e.g. lack of knowledge, infrastructure), cultural (e.g. it does not fit existing norms), institutional (e.g. obstructive laws and regulations) or economic (e.g. negative externalities not included in the market price) (Kirchherr et al. 2018). However, some barriers may be easier to overcome than others. To make the fourth characteristic more concrete, a transformative initiative should have overcome a barrier that other actors have not been able to overcome. Within the specific context of the initiative it was possible to overcome this barrier by collective effort, creating conditions, seizing opportunities or altering constraints. When it comes to assessing the transformative potential of initiatives in terms of the fourth small win characteristic, we distinguish between three categories of initiatives (ordered from least to most transformative potential): (1) initiatives that did not encounter any significant barriers in the development of the initiative (2) initiatives that have been able to overcome one or more barriers that were relatively easy to overcome and (3) initiatives that have been able to overcome one or more barriers that were relatively hard to overcome.

The illustrative initiatives have overcome barriers to different extents. In the case of *Fertile Nutrient Cycles*, most of the measures implemented by farmers did not face many barriers. Because most of the measures increase input–output efficiency, they even benefitted the participating farmers economically. The initiative of *Circular Broilers* has overcome some relatively hard barriers, as it had to find a new revenue model to compensate for increased costs. By cooperating with a meal box company, the initiative was able to ensure a more stable income. *Agricycling* has faced barriers that were possibly the hardest to overcome, as the recycling of waste streams was initially not allowed under existing legislation. Local officials have granted exemptions for these regulations to support the initiative.

### Synergy technological and social change

In a transition to circular agriculture, social change is as important as technological change. For example, the re-using and recycling of materials and substances does not only imply other agricultural practices but also new collaborations between actors in the food chain (Fischer and Pascucci 2017) and an integration of tasks instead of linear specialisation (as in the case of reintegrating plant and livestock systems, see Garrett et al. 2020). Conserving and regenerating the health of agro-ecosystems (safeguard principle) requires new ways of value creation, rules and incentives (Vermunt et al. 2020). Moreover, halting overconsumption and food waste (prevent principle) implies a shift of norms and social practices among consumers and businesses (Schanes et al. 2018). We define social change as *‘changes in social practices and relations involving new ways of doing, organising, knowing and framing’* (Avelino et al. 2019). What changes with social innovation is *‘the way how people decide, act and behave, alone or together’* ((Franz et al. 2012) in Avelino et al. 2019). In the context of circular agriculture, it may include new collaborations, business models, consumer-producer relations, certification, regulations and standards. We distinguish three levels of the interaction between social and technological change. First, an initiative may be (almost) purely technological or social. An example is an emission-reducing stable, which requires a minimal adjustment of social practices. Second, an initiative may be dominantly technological, enabled by forms of social innovation (or vice versa). An example is extensive dairy farming enabled by a new consumer label. The most transformative level is when synergy between social and technological change occurs. An example would be community-supported agriculture, in which the technological innovation could hardly exist without the social innovation, and vice versa.

All initiatives show a connection between societal and technological change, but to different extents. In the case of *Fertile Nutrient Cycles,* the main focus is on technological innovation, which is supported by social innovations such as a platform to share knowledge about techniques between farmers. Although the innovation in the case of *Circular Broilers* is primarily technological (recycling waste streams as feed), it is strongly enabled by a novel cooperation with neighbouring farms and the meal box company. In the case of *Agricycling*, both the technological and social changes are profound and strengthen each other. The farmers do not only recycle municipal waste streams as fertilisers but have organised themselves in a cooperative, creating a new source of income and showing a level of professionalisation that attracts support from policymakers. This institutionalisation makes the initiative more robust. The regulatory exemptions that are granted to this initiative have been copied by other municipalities, enabling similar initiatives to emerge in other regions.

Box 1. Three circular agriculture initiatives We selected three illustrative initiatives from the Netherlands, as the Dutch government aims to be a world leader in circular agriculture (Ministry of Agriculture, Nature and Food Quality of the Netherlands 2018). These initiatives were selected for their demonstrated contribution to circular agriculture and recognition as potential solutions. For each initiative, we did a preliminary case analysis based on publicly available resources.

*Vruchtbare kringloop Noord-Nederland* (referred to as: ‘fertile nutrient cycles’) aims to improve the nitrogen and phosphorus cycles at dairy farms in the North-Netherlands by reducing nutrient losses, avoiding emissions and saving inputs. To do so, the project encourages improved manure and feed practices, such as the addition of water when spreading manure on the land to avoid emissions, outside grazing to increase manure efficiency, optimization of the feed protein content, better timing of manure application, and encouraging natural nitrogen fixation, amongst others.

*Oranjehoen* (referred to as ‘circular broilers’) is a mixed arable and broiler farm. The arable farm produces organic food crops like carrots, of which usually five to ten percent are rejected for sale because of quality reasons. The farm cooperates with neighbouring arable farms to recycle this residual stream as feed for the broilers, thereby replacing feed concentrates. The manure of the broilers is returned to the land as fertiliser. To ensure continuous income and to compensate for higher operating costs, the farm sells the meat to i.a. a meal box company.

*Agricycling*, a cooperative of twelve farmers, aims to substitute synthetic fertilizers with composted municipal waste streams. The farmers collect and compost roadside clippings and sludge, residual streams that contain valuable nutrients but are normally treated as waste. By substituting synthetic fertilizer, this approach saves emissions (carbon oxide and nitrate), sequesters carbon and enhances soil life, while generating a new business model as the farmers are paid for the recycling. As farmers have no permission to process waste streams within current regulatory frameworks, the cooperative has been granted exemptions. In the future, the cooperative aims to close the cycle of food production and consumption by reusing food waste and human excreta.

## Discussion and conclusion

The aim of this paper was to conceptualise the characteristics that determine the transformative potential of circular agriculture initiatives. To answer this question, we further advanced the small wins characteristics and specified these to the context of circular agriculture. Instead of treating the small wins characteristics as binary attributes, we acknowledge that initiatives can exhibit these characteristics to varying degrees, indicating different levels of transformative potential. For each small win characteristic, we therefore conceptualised different levels (e.g. improve, reform and redesign for the depth of change).

While there has been a sprawl of agricultural initiatives that are labelled as ‘circular’, the heuristic provides a way of scrutinizing the degree to which initiatives may truly induce transformative change (see Table 3). For example, we showed that the illustrative case of *Fertile Nutrient Cycles* contributes to circular principles mainly through adjustments of existing practices. While these first order changes may reduce the environmental impact of farming, the initiative mainly optimises the existing production model that still relies on the use of synthetic fertilizer and feed concentrates. Additionally, the changes are primarily technological and did not cause resistance or any major barriers. In that sense, the initiative may not be considered a small win yet, but as the initiative shows concrete results and encourages learning, it may become one if it would address problems at a deeper level.

Compared to *Fertile Nutrient Cycles*, the initiative of *Circular Broilers* appeared to have more transformative potential as it breaks through the trend of using imported feed concentrates to raise broilers. By establishing novel cooperations with neighbouring arable farms to recycle crop residues as feed for the broilers, this initiative changes the way that goals are achieved and could therefore be characterized as a second-order change. *AgriCycling* could be characterized as bringing about the most profound change, as it aims to fundamentally restructure the flows of nutrients by reusing municipal waste streams to replace synthetic fertilizers, thereby addressing problems at their root cause.

As shown by the above examples, the heuristic can function as a tool to foster debates among policymakers and stakeholders to evaluate the extent to which initiatives challenge the linear agricultural paradigm along their contribution to circular principles, the concreteness of results, the depth of change, experienced barriers and connection between technical and social change. The relative importance of the characteristics and the extent to which initiatives should meet the characteristics depends upon the ambitions and challenges that policymakers and other stakeholders face within a given context. It is also important to note that the transformative potential of initiatives may change over time, as initiatives may continuously reinvent and adapt themselves (Schagen et al. 2022). In that regard, the heuristic can help to identify opportunities, develop strategies and monitor the development of initiatives.

Although our aim is not to prescribe a critical level of transformative potential beyond which initiatives should be supported, we argue that given the environmental challenges and policymakers’ aspirations for circularity, it is wise to support initiatives that foster circularity through second or preferably third order changes. While first order changes may be an initial step towards circularity and more feasible to scale, mere adjustments or efficiency improvements are not sufficient to address the environmental challenges of our food systems (Conijn et al. 2018). Additionally, since first-order changes optimise existing practices, there is a risk that supporting first-order initiatives makes it even more difficult and costly to eventually realize more transformative change, thereby reinforcing a ‘lock-in’ of the status quo (Seto et al. 2016). For example, encouraging livestock farmers to invest in emission-reducing stables, one of the policy proposals to reduce nitrogen emissions in the Netherlands, actually reinforces farmers’ dependence on their current way of farming, thereby limiting possibilities to address environmental problems at their root cause instead (Vink et al. 2021). For that reason, before replicating and expanding first order initiatives, these initiatives would benefit from increasing their transformative potential first.

To realize the full potential of circular agriculture initiatives, the role of policymakers is not limited to identifying and appreciating transformative initiatives. As shown by the example of *AgriCycling*, more transformative initiatives are likely to face more economic and institutional barriers that cannot be overcome by the initiatives themselves. The spreading, broadening and deepening of circular agriculture initiatives will also depend on developments in the surrounding food system, including that of consumer behaviour, retail markets, input suppliers and financing, as well as broader economic, political and biophysical drivers (cf. HLPE 2017). Policymakers can play a vital role in creating the right rules and incentives across these systems to accelerate transformative change (Ruben et al. 2021). This includes the reform of existing policies, such as the EU's Common Agricultural Policy (CAP), which currently provides direct payment subsidies to support status quo farming practices, rather than incentivizing more circular practices (Buitenhuis et al. 2020).

Beside furthering the debate on circular agriculture, our conceptual argument is also of relevance to the broader transformation literature, in two ways. First, observing that the characteristics of transformative initiatives remain insufficiently and inexplicitly described in the transformation literature, we proposed to conceptualize this transformative potential as a function of five distinct characteristics, enabling scholars to discuss more explicitly what it is that makes initiatives more or less transformative. Second, although there is a lot of attention for the amplification and scaling of initiatives (e.g. Gorissen et al. 2018; Lam et al. 2022), scholars have so far mostly avoided the more normative question of which initiatives ought to be scaled (Pitt and Jones 2016; El Bilali 2019). We argue that this question deserves more attention, which is why in this paper we articulate what types of initiatives hold the most potential for transformative change in the context of circular agriculture. We hope that our paper will be a starting point for wider discussions on what types of initiatives deserve support, within the context of sustainable agriculture and beyond.

In this sense, although we used examples from the Dutch context to illustrate our conceptual argument, we believe the heuristic has analytical value in other contexts as well, including the Global South. While different contexts require tailored solutions, circular principles, such as avoiding waste and recycling, have universal potential to guide the use of resources within planetary boundaries (Ruben et al. 2021). To fully leverage the potential of emerging circular initiatives across various contexts, it is important to stimulate initiatives that have concrete results, are in-depth, faced barriers and integrate technological and social change. Specific examples of initiatives that exhibit these features however depend on local agro-ecological and socio-economic conditions. The heuristic may be used for other societal challenges as well, such as the energy transition, by adjusting the principles accordingly.

We see various avenues for future research. The main objective of this research was to advance the characteristics of transformative initiatives conceptually. A next step in the development of the heuristic would be to apply it more systematically to a set of initiatives to test its empirical applicability. Future research is required to answer the question of what conditions are needed for circular agriculture initiatives to actually fulfil their transformative potential.

To conclude, emerging circular initiatives could be the seeds for much-needed transformative change in agriculture, but to varying degrees. Introducing this heuristic, we can better identify which initiatives truly have the potential for a transformation towards a more circular agriculture.
